# SEC24A identified as an essential mediator of thapsigargin-induced cell death in a genome-wide CRISPR/Cas9 screen

**DOI:** 10.1038/s41420-018-0135-5

**Published:** 2018-12-18

**Authors:** Tamutenda Chidawanyika, Elizabeth Sergison, Michael Cole, Kenneth Mark, Surachai Supattapone

**Affiliations:** 1 Department of Biochemistry, Geisel School of Medicine at Dartmouth, Hanover, NH 03755 USA; 2 Department of Molecular and Systems Biology, Geisel School of Medicine at Dartmouth, Hanover, NH 03756 USA; 3 Norris Cotton Cancer Center, Geisel School of Medicine at Dartmouth, Hanover, NH 03756 USA; 4 Department of Medicine, Geisel School of Medicine at Dartmouth, Hanover, NH 03755 USA

## Abstract

Endoplasmic reticulum (ER) stress from accumulated misfolded proteins in the ER can activate the unfolded protein response (UPR). The UPR acts either to restore proteostasis or to activate cell death pathways if the stress cannot be resolved. The key downstream effectors in these pathways have been studied extensively. However, in comparison, stressor-specific key mediators are not as well characterized. In this study, we sought to identify and compare the genes that are necessary for cell death induced by three classic pharmacological ER stressors with different mechanisms of action: thapsigargin, tunicamycin, and brefeldin A. We conducted genome-wide CRISPR/Cas9-based loss-of-function screens against these agents in HAP1 cells, which are a near-haploid cell line. Our screens confirmed that MFSD2A and ARF4, which were identified in previous screens, are necessary for tunicamycin- and brefeldin A-induced cytotoxicity, respectively. We identified a novel gene, SEC24A, as an essential gene for thapsigargin-induced cytotoxicity in HAP1 cells. Further experiments showed that the ability of SEC24A to facilitate ER stress-induced cell death is specific to thapsigargin and that SEC24A acts upstream of the UPR. These findings show that the genes required for ER stress-induced cell death are specific to the agent used to induce ER stress and that the resident ER cargo receptor protein SEC24A is an essential mediator of thapsigargin-induced UPR and cell death.

## Introduction

The accumulation of misfolded proteins in the endoplasmic reticulum (ER) results in ER stress. To alleviate the ER stress, the unfolded protein response (UPR) is activated. Depending on the degree of cellular damage, the UPR acts to either restore homeostasis and rescue the cell or to kill the cell through tightly regulated cellular death pathways, such as apoptosis^1,2^.

ER stress can be attained by disturbing components of the ER machinery. Pharmacologically, this can be achieved by treating cells with classic ER stressors, such as tunicamycin, brefeldin A, and thapsigargin, all of which use distinct mechanisms of action to perturb the ER. Tunicamycin inhibits UDP-GlcNAc:dolichol phosphate GlcNAc-1-phosphate transferase (DPAGT1), an enzyme that is important for one of the first steps in asparagine (N)-linked glycosylation of proteins in the ER lumen^3,4^. Inhibition of this process results in protein misfolding and, subsequently, ER stress^5^. Brefeldin A perturbs ER–Golgi protein trafficking through its interactions with ADP-ribosylation factors (ARFs), which are important for cargo transport between the ER and Golgi^6–8^. As a consequence of this perturbance, ER stress ensues due to disrupted protein secretion and collapse of the Golgi into the ER^9^. Thapsigargin upsets calcium homeostasis in the ER by inhibiting sarcoplasmic/endoplasmic reticulum Ca^2+^ ATPase (SERCA) pumps^10,11^. The consequent depletion of calcium stores in the ER lumen compromises the functions of calcium-dependent chaperones in the ER resulting in protein misfolding^10^.

The use of these agents as biochemical tools has advanced our understanding of ER stress and protein trafficking. More recently, these agents have been used to study ER stress-induced cell death. The use of gene trap mutagenesis in haploid genetic screens has allowed for the identification of some of these necessary cell death mediators that act when cells are exposed to specific ER stressors. A screen performed in KBM7 cells, which are near-haploid cells, for mediators of tunicamycin-induced cell death identified MFSD2A (major facilitating domain 2A), a plasma membrane transporter^3^, as critical, whereas a similar screen for mediators of brefeldin A-induced death identified ARF 4 (ARF4)^6^ as critical.

Since the findings from the tunicamycin and brefeldin A screens indicated that the key mediators necessary for ER stress-induced cell death to be carried to completion were specific to the nature of the initial insult to the ER, we sought to explore this idea further. In this study, we use pooled CRISPR/Cas9 human libraries to conduct comprehensive and unbiased loss-of-function screens against thapsigargin, tunicamycin, and brefeldin A in a single-cell type, HAP1 cells, to identify and compare genes necessary for induction of cell death by these agents.

We found that the genes required for ER stress-induced cell death are specific to the agent used to induce ER stress and that SEC24A is an essential mediator of thapsigargin-induced UPR and cell death.

## Results

### Genes identified from positive selection screens against thapsigargin, tunicamycin, and brefeldin A

To identify and compare genes that are necessary for cell death induced by thapsigargin, tunicamycin, and brefeldin A, positive selection screens were conducted in CRISPR/Cas9-modified HAP1 cell libraries using each of the three compounds to induce ER stress and cell death. Screens were conducted at concentrations that resulted in <1% cell survival determined from cytotoxicity curves generated for each compound in HAP1 WT cells (Supplementary Fig. 1). The selected concentrations were: thapsigargin, 0.062 µg/mL; tunicamycin, 0.2 µg/mL; and brefeldin A, 0.045 µg/mL. The CRISPR/Cas9-modified HAP1 cell libraries were generated by transducing HAP1 WT cells with 2 lentiviral sgRNA libraries (A and B) designed to target 19,050 genes in total. Within the library, each gene was targeted by six unique sgRNAs. All three of the screens yielded surviving cells after four rounds of selection. The DNA from these cells was isolated and deep sequenced to identify the genes represented in the enriched mutant populations.

**Fig. 1:**
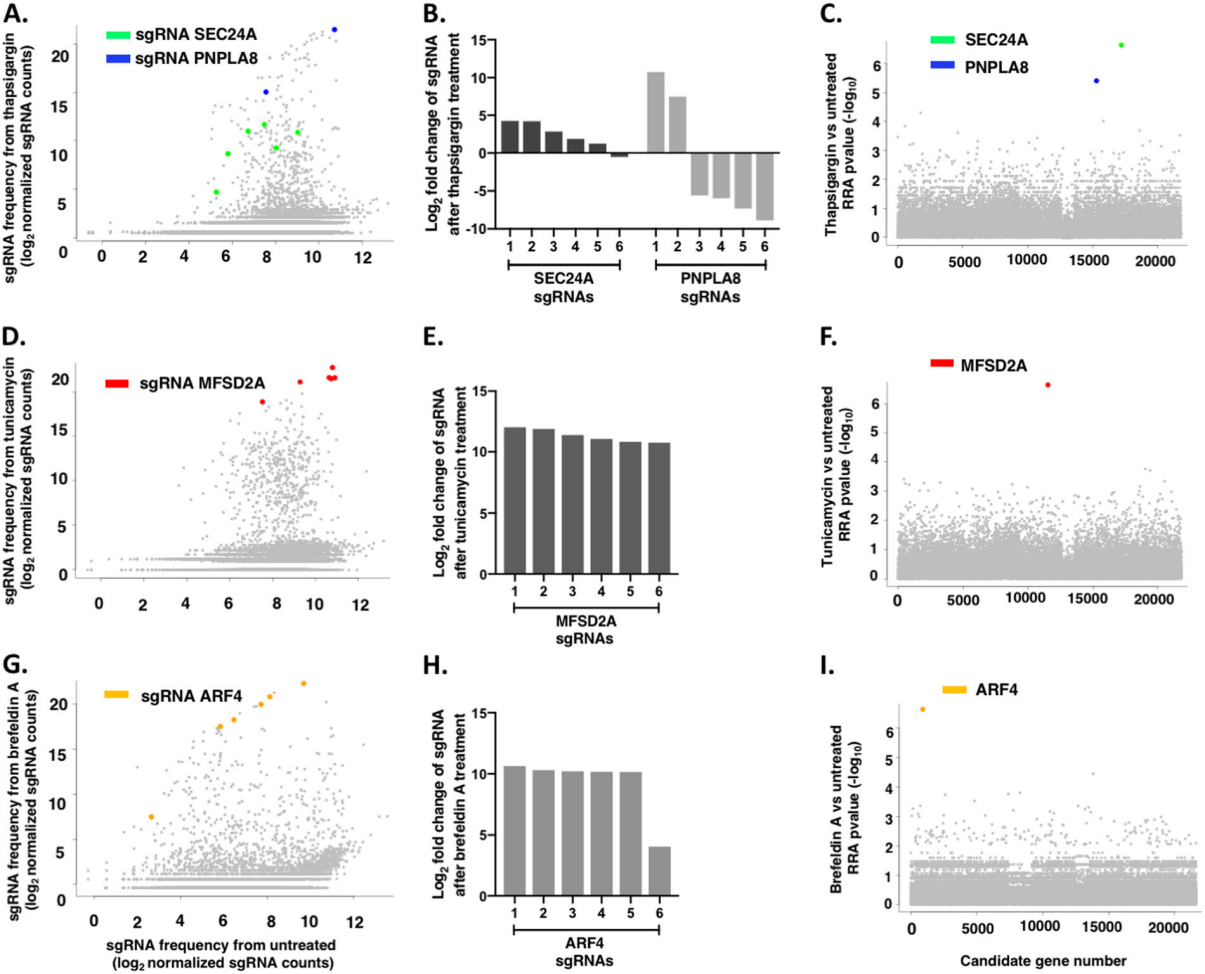
Genes isolated from positive selection screens in CRISPR/Cas9- modified HAP1 cells using ER stressors. Each gene is targeted by a total of six sgRNAs; therefore, a screen showing a robust survival phenotype will show multiple sgRNAs targeting the same gene. Scatterplots showing the enrichment of sgRNAs in cells treated with **a** thapsigargin (0.062 µg/mL), **d** tunicamycin (0.2 µg/mL), and **g** brefeldin A (0.045 µg/mL), compared to the representation of these sgRNAs in untreated cells. **b**, **e**, **h** Bar graphs showing fold changes of sgRNAs targeting candidate genes after compound treatment relative to untreated cells. **c**, **f**, **i** Identification of top candidate genes in each screen using RRA *p* value from MAGeCK analyses

The thapsigargin screen identified two novel candidate genes, SEC24A and PNPLA8 (patatin-like phospholipase containing domain 8) (Fig. 1a). SEC24A encodes for a component of the coat protein complex II (COPII), which is important for ER–Golgi protein trafficking^12–14^, while PNPLA8 encodes for a calcium-independent phospholipase^15^. The strength of the SEC24A hit is reflected in the fact that five out of six of the sgRNAs targeting SEC24A showed high frequencies of occurrence and increases in sgRNA representation in cells treated with thapsigargin compared to untreated cells (Fig. 1a, b). This indicates that these sgRNAs were enriched in cells treated with thapsigargin compared to these same sgRNAs in untreated cells. Using MAGeCK, a robust ranking aggregation (RRA) algorithm was used to rank candidate genes based on the degree of consistency seen in enrichment of multiple sgRNAs targeting a specific gene, and significance scores, through *p* values, were assigned for each gene^16^. Lower RRA scores indicate genes of importance. The low RRA score for SEC24A (8.14 × 10^−15^) (Table 2), as well as the low *p* value generated for this RRA score (2.27 × 10^−7^) (Table 2, Fig. 1c), are consistent with the count data, which showed enrichment of five of the six sgRNAs targeting SEC24A in library cells treated with thapsigargin. These data show that SEC24A was a true hit in this screen. Similar to SEC24A, PNPLA8 was highly ranked as a candidate gene for the thapsigargin screen. However, only two out of six of the sgRNAs targeting PNPLA8 showed strong enrichment in cells treated with thapsigargin compared to untreated cells (Fig. 1a, b). PNPLA8 had a much higher RRA score (1.74 × 10^−6^) than SEC24A (8.14 × 10^−15^) (Table 2), consistent with the count data, indicating that PNPLA8 was a less compelling candidate gene than SEC24A in this screen. Thus, although the RRA score for PNPLA8 was significant (*p* = 3.86 × 10^−6^) (Table 2, Fig. 1c), the lack of enrichment by 4 of the 6 sgRNAs suggested that PNPLA8 may not be a true hit.

In the tunicamycin screen, six out of six of the sgRNAs targeting MFSD2A, a gene that encodes for a transmembrane protein^3,17^, showed high frequencies of occurrence and increases in sgRNA representation in cells treated with tunicamycin compared to untreated cells (Fig. 1d, e). Further, MAGeCK analysis ranked MFSD2A as an important gene in the tunicamycin screen as shown by a low RRA score (6.27 × 10^−26^, *p* = 2.27 × 10^−7^) (Fig. 1f, Table 3).

Similarly, the brefeldin A screen identified a single strong hit in ARF4, as 6 out of 6 of the sgRNAs targeting ARF4 showed high frequencies of occurrence and increases in sgRNA representation in cells treated with brefeldin A compared to untreated cells (Fig. 1g, h). MAGeCK analysis produced a low RRA score (1.05 × 10^−19^, *p* = 2.29 × 10^−7^) (Fig. 1i, Table 4).

Both MFSD2A and ARF4 have been previously identified in KBM7 cells as being necessary for tunicamycin- and brefeldin A-induced cell death, respectively, using gene trap mutagenesis screens^3,6^. The identification of these genes in our screens against the same agents confirms the prior results and demonstrates the reliability of our CRISPR/Cas9-based screening methodology.

### Loss of function of SEC24A results in resistance to thapsigargin

**Fig. 2:**
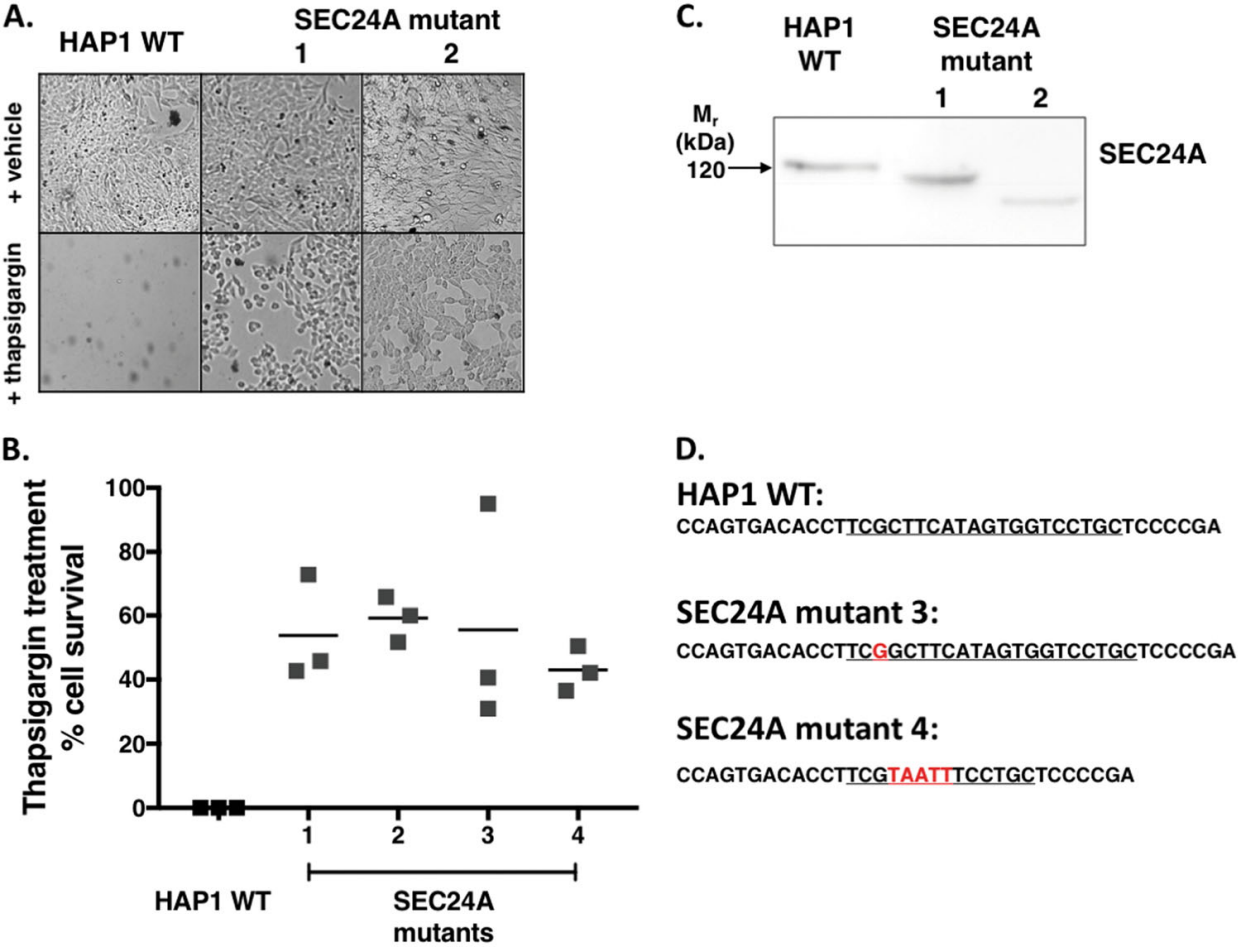
Validation of SEC24A candidate gene from thapsigargin screen. Four monoclonal SEC24A mutant cell lines were generated from two sgRNAs. SEC24A mutant 1 and SEC24A mutant 2 were generated from SEC24A sgRNA 1 (Table 1), and SEC24A mutant 3 and SEC24A mutant 4 were generated from SEC24A sgRNA 2 (Table 1). **a** After 3 days of treatment with 0.062 µg/mL thapsigargin, SEC24A mutant cell lines confer resistance to thapsigargin-induced cell death compared to HAP1 WT cells. **b** Quantitative representation of **a**. After 3 days of treatment with 0.062 µg/mL thapsigargin, cell survival was assessed in SEC24A mutant cell lines and HAP1 WT cells. *N* = 3. **c** Western blot showing SEC24A protein in HAP1 WT cells and representative SEC24A mutants. SEC24A protein was modified in both SEC24A mutant 1 and SEC24A mutant 2 monoclonal cell lines as shown by the shift in molecular weight compared to SEC24A protein in HAP1 WT cells. **d** Genotyping of representative SEC24A mutants. The region of interest in the SEC24A gene targeted by the sgRNA showed insertion mutations in both SEC24A mutant 3 and SEC24A mutant 4. Underlined regions indicate the location within the SEC24A gene targeted for mutation by SEC24A sgRNA 2. Insertion mutations resulting in frameshifts and subsequently in stop codons are shown in red

To validate that SEC24A was necessary for thapsigargin-induced cell death, HAP1 WT cells were transfected with a lentiCRISPRv2 vector containing sgRNAs targeting SEC24A (SEC24A sgRNA 1 and SEC24A sgRNA 2 in Table 1). Two monoclonal SEC24A mutant cell lines were isolated from each sgRNA by serial dilution (SEC24A mutants 1 and 2 from SEC24A sgRNA 1, and SEC24A mutants 3 and 4 from SEC24A sgRNA 2). When exposed to 0.062 µg/mL thapsigargin for 3 days, all of the SEC24A mutant cell lines conferred resistance to thapsigargin-induced cell death, as shown by the high percentage of cell survival in the SEC24A mutants compared to the HAP1 WT control cells (Fig. 2a, b). To confirm that these phenotypic observations were due to mutations in SEC24A, western blots and genotyping were performed on the SEC24A mutant monoclonal cell lines. For western blots, lysates from SEC24A mutant 1 and SEC24A mutant 2 monoclonal cells were analyzed for SEC24A protein by probing with a polyclonal antibody that recognizes epitopes located N-terminal to the sgRNA target region. The shift in molecular weight for SEC24A seen in the mutant cell lines compared to HAP1 WT cells indicates that resistance to thapsigargin observed in mutant cell lines was associated with frameshift mutations in the SEC24A protein (Fig. 2c). For genotyping, the sgRNA ROI in the SEC24A gene was analyzed in SEC24A mutant 3 and SEC24A mutant 4 monoclonal cell lines. In both SEC24A mutants 3 and 4, insertions resulting in frameshift mutations and stop codons occurred (Fig. 2d), confirming that the thapsigargin-resistance phenotype seen in SEC24A mutants was due to loss of function of SEC24A.

To validate that PNPLA8 was necessary for thapsigargin-induced cell death, two monoclonal PNPLA8 mutant cell lines were generated from an sgRNA targeting PNPLA8 (PNPLA8 sgRNA 1 in Table 1). These cell lines also exhibited resistance to thapsigargin as shown by the significantly greater cell viability seen in the mutant cell lines compared to the control cell line after 3 days of treatment (Supplementary Fig. 2A). However, the observed thapsigargin-resistance phenotype could not be attributed to mutations in PNPLA8. Western blot analysis failed to identify any modifications in the PNPLA8 gene in the mutant cell lines when compared to HAP1 WT cells (Supplementary Fig. 2B), and no mutations at the nucleic acid level were found in the PNPLA8 mutants when compared to control HAP1 WT cells (Supplementary Fig. 2C). These findings indicated that PNPLA8 was not the gene responsible for the observed phenotype and therefore was not a valid hit in our screen. The PNPLA8 data suggest an off-target effect of the sgRNA.

### The required genes for ER stress-induced cell death are stressor specific

Because the results of our screens indicated that each of the three ER stressors required different genes to induce cell death in HAP1 cells, we sought to determine whether the genes necessary for resistance to ER stress-induced cell death were stressor specific. Multiple mutant cell lines were generated for each candidate gene identified in our screens using multiple unique sgRNA sequences (Table 1). For SEC24A, three polyclonal mutant cell lines were generated using transfection methods. For MFSD2A and ARF4, two polyclonal mutant cell lines were generated. A non-targeting negative control cell line was generated using an sgRNA against enhanced green fluorescent protein (Table 1). These cell lines were then each treated with thapsigargin, tunicamycin, or brefeldin A for 3 days.

**Fig. 3:**
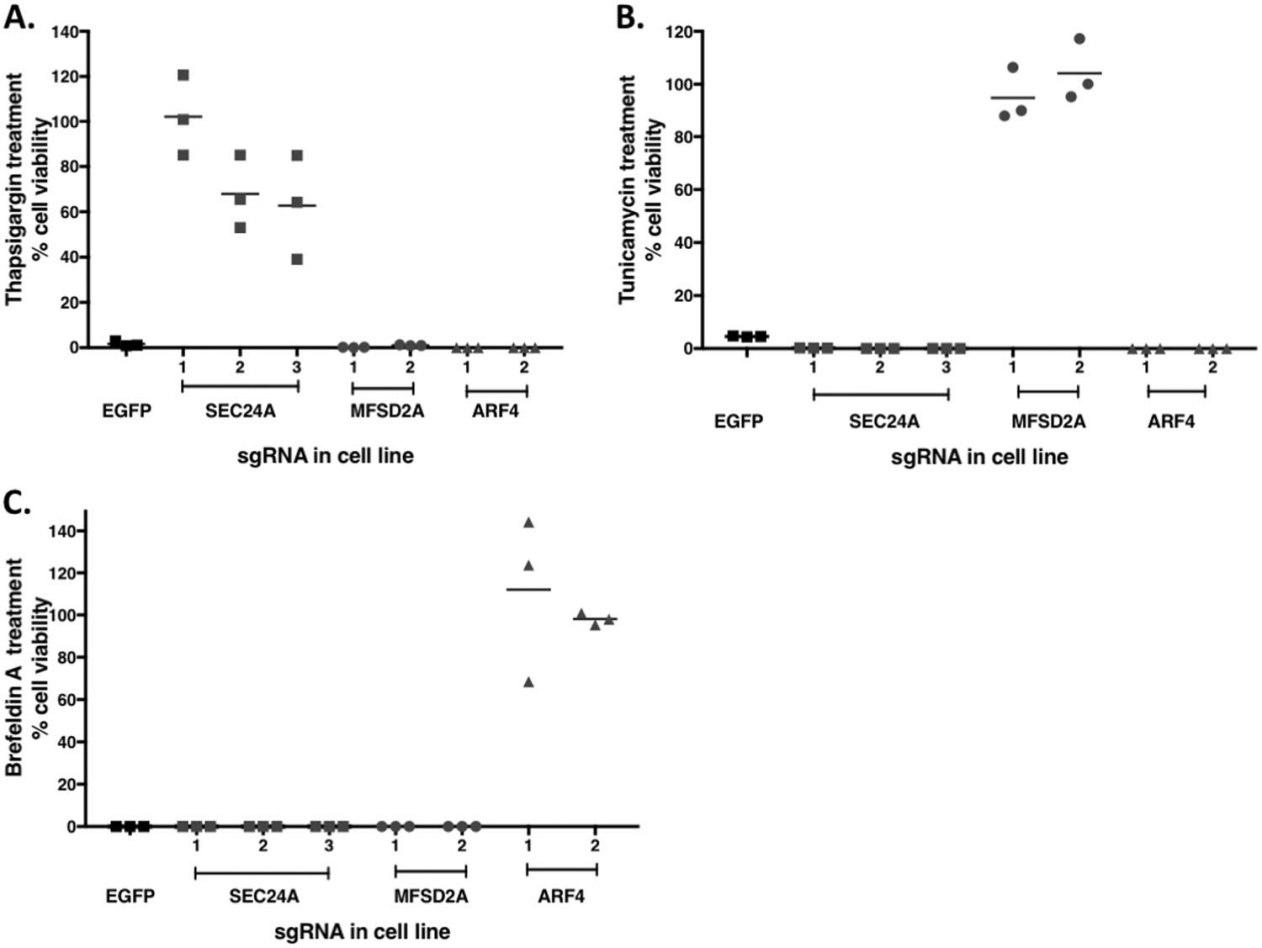
Genes that are necessary for ER stress-induced cell death are specific to the agent of stress. Mutant polyclonal cell lines (three SEC24A mutant cell lines from three different sgRNAs, two MFSD2A mutant cell lines from two different sgRNAs, and two ARF mutants from two different sgRNAs, all shown in Table 1) were treated with the ER stressors used in the screens and assessed for cell survival using trypan blue. SEC24A, MFSD2A, and ARF mutant cell lines were assessed for cell survival after 3 days of treatment with **a** thapsigargin (0.062 µg/mL), **b** tunicamycin (0.2 µg/mL), or **c** brefeldin A (0.045 µg/mL). Cells transfected with EGFP were the non-targeting negative control for all treatments with ER stressors. *N* = 3 for each cell line and for each of the treatments

When SEC24A mutants were treated with thapsigargin, all of the cell lines showed significant survival compared to the non-targeting control cell line. At least 40% of the cells in each of the three SEC24A mutant cell lines survived drug treatment relative to untreated SEC24A mutants, while in the control cell line, only ~1% of the cells survived (Fig. 3a). In direct contrast, when MFSD2A and ARF4 mutants were treated with thapsigargin, the cells did not survive the treatment, and their percentage of survival, relative to their untreated mutants, was similar and comparable to the non-targeting control cells (Fig. 3a). Upon treatment with tunicamycin, the MFSD2A polyclonal mutants showed significant resistance to tunicamycin-induced cytotoxicity compared to the non-targeting control cell line, with almost 100% of the mutants surviving treatment (Fig. 3b). However, when SEC24A and ARF4 mutants were treated with tunicamycin, the cells did not survive (Fig. 3b). Lastly, brefeldin A treatment resulted in almost no sensitivity in ARF4 polyclonal mutants, where close to 100% of the cells survived the treatment, while 100% of the non-targeting control cells died (Fig. 3c). However, brefeldin A treatment induced significant cell death in SEC24A and MFSD2A polyclonal mutants.

Cumulatively, these data indicate that the essential facilitators for ER stress-induced cell death are specific to the pharmacological agent used to induce ER stress. Additionally, since thapsigargin, tunicamycin, and brefeldin A can all cause ER stress through different mechanisms of initial insult, these data suggest that the essential mediators are specific to the nature of the initial insult to the cell caused by these agents.

### In SEC24A mutants, the UPR is functional but only slightly activated by thapsigargin

ER stress induction results in activation of the UPR in HAP1 WT cells. In principle, SEC24A could act either upstream or downstream of the UPR. To evaluate the role of SEC24A in UPR activation, two SEC24A mutant monoclonal cell lines (SEC24A mutants 1 and 2) were treated with thapsigargin, tunicamycin, or brefeldin A at screening concentrations for 18 h and then analyzed for the levels of two markers of UPR activation, CHOP and ATF4.

**Fig. 4:**
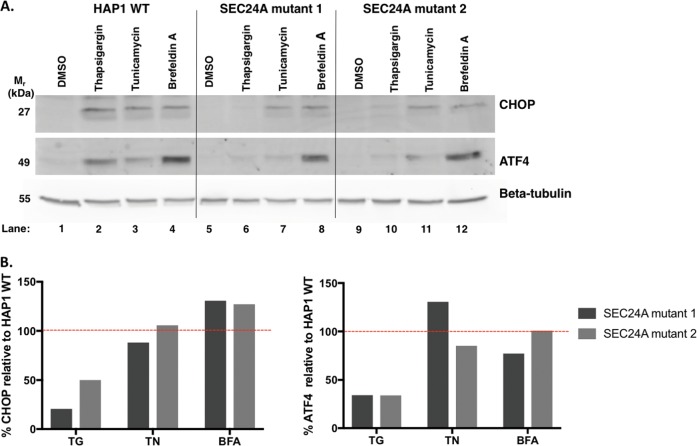
Thapsigargin-induced UPR is diminished in SEC24A mutants. CHOP and ATF4 levels were used as indicators of UPR activation in HAP1 WT and representative SEC24A mutants 1 and 2 after an 18 h treatment with thapsigargin (TG; 0.062 µg/mL), tunicamycin (TN; 0.2 µg/mL), or brefeldin A (BFA; 0.045 µg/mL). **a** Western blot of CHOP and ATF4 as indicators of the UPR in HAP1 WT cells and SEC24A mutants with and without ER stress induction. **b** Quantification of CHOP and ATF4 levels in SEC24A mutants relative to HAP1 WT cells for each treatment. Levels are expressed as percentages, where 100% (dotted line) represents CHOP or ATF4 levels in treated HAP1 WT cells

To determine whether the UPR was activated in HAP1 WT cells by thapsigargin, tunicamycin, or brefeldin A, CHOP and ATF4 levels in HAP1 WT cells after the different treatments were compared to CHOP and ATF4 levels in untreated HAP1 WT cells. As shown by the significant increases in band intensities of CHOP and ATF4, thapsigargin, tunicamycin, and brefeldin A all activated the UPR in HAP1 WT cells (Fig. 4a, lanes 2–4). Based on ATF4 levels in HAP1 WT cells, brefeldin A was the strongest activator of the UPR (lane 4), followed by thapsigargin (lane 2), and then tunicamycin (lane 3) (Fig. 4a). These data suggest that different agents activate the UPR to different degrees in HAP1 WT cells.

Treatment with the different ER stress agents activated the UPR in the SEC24A mutants, shown by increases in CHOP and ATF4 compared to untreated mutants (Fig. 4a; compare lanes 6–8 to lane 5, and lanes 10–12 to lane 9). Similar to HAP1 WT cells, in SEC24A mutants the different agents activated the UPR to different degrees. Notably, however, when treated with thapsigargin, CHOP and ATF4 levels in SEC24A mutants were reduced to <50% of the levels observed in treated HAP1 WT cells (Fig. 4b, lanes 6 and 10). Tunicamycin or brefeldin A treatments of SEC24A mutants resulted in CHOP and ATF4 levels that were comparable to the corresponding levels in treated HAP1 WT cells (Fig. 4b). Together, these data indicate that the UPR is functional in SEC24A mutants and that the inability of SEC24A mutants to robustly activate the UPR is specific to thapsigargin. Additionally, the findings suggest that, with regard to thapsigargin-induced cell death, SEC24A acts upstream of the UPR.

## Discussion

In this study, we used CRISPR/Cas9 libraries to conduct positive selection screens against thapsigargin, tunicamycin, and brefeldin A, which are classic ER stressors with different mechanisms of action. Our goal was to identify and compare genes that are essential for cell death to occur as a result of these agents. Genome-wide positive selection screens using tunicamycin and brefeldin A have been conducted previously in KBM7 cells using gene trap mutagenesis techniques^3,6^. The HAP1 cells used in our screens were derived from KBM7 cells^18^. Similar to these previous screens, our screens with tunicamycin and brefeldin A yielded MFSD2A and ARF4, respectively, as being necessary for cell death to occur. These confirmatory results serve as indicators of the reliability of our screening methodology with regard to ER stress-induced cell death. More importantly, these results suggest that the screens for tunicamycin and brefeldin A were saturated. The thapsigargin screen identified a novel gene, SEC24A, as a necessary mediator of thapsigargin-induced cytotoxicity. The survival of SEC24A mutant cells after treatment with thapsigargin when HAP1 WT cells did not survive validated the results of our thapsigargin screen.

Thapsigargin, tunicamycin, and brefeldin A can all induce ER stress, which if unresolved, can result in cell death. Although we found that each of these agents can result in ER stress-induced cell death in HAP1 WT cells, our screens did not identify any shared genes across the different treatments that were necessary for induction of cell death. A simple interpretation of our data is that no single ER stress-induced cell death pathway exists, since no common genes were found to be necessary when different ER stressors were used to induce cell death. However, it is entirely possible that there is redundancy in gene function and cell death pathways that can be employed by the different agents. Redundant genes would not be detected in positive selection loss-of-function screens. Lastly, it is possible that common pathway genes are all essential for cell viability. Screening for essential genes is not possible in a loss-of-function screen.

These last two interpretations highlight some of the strengths and limitations of our screens. Using near-haploid cells and CRISPR/Cas9-mediated mutagenesis increased the likelihood of generating cells with full gene knockouts in our screens. Even though this is a advantage for determining the necessary mediators for a particular process, we cannot identify key players that are either essential or redundant. Two pertinent examples that further emphasize the strengths and limitations of our screening strategy are shown by a previous siRNA screen to identify mediators of thapsigargin-induced cytotoxicity and a recent CRISPR-based transcriptional interference (CRISPRi) screen to explore the genes involved in ER homeostasis after tunicamycin or thapsigargin treatment^19^. The siRNA screen was conducted in HCT116 cells, which are near-diploid colon cancer cells, and the results identified a network of antiapoptotic genes as being involved in thapsigargin-induced cell death^20^. In the CRISPRi screen conducted in near-triploid K562 cells, multiple mediators that play roles in ER homeostasis and UPR activation after tunicamycin or thapsigargin treatment were identified^19^. SEC24A was not among the identified genes in either of these screens. siRNA and CRISPRi screens allow for some protein function to be retained, and therefore, all of the contributing proteins to a process can be identified. However, necessary mediators that can only be identified from a complete loss of function of the gene cannot be identified. Additionally, the use of diploid or polyploid cells such as HCT116 and K562 cells, rather than a near-haploid cell line such as HAP1 cells, would allow for the identification of more mediators involved in a process if the genome modifications used were not complete gene knockouts. Our screening approach, however, sought to identify the essential mediators for thapsigargin-induced cell death, and in HAP1 cells, the only essential mediator of thapsigargin-induced cell death is SEC24A.

Thapsigargin, tunicamycin, and brefeldin A can all activate the UPR in HAP1 WT cells as shown by increases in CHOP and ATF4 levels. CHOP is a known mediator of apoptosis in the UPR and ATF4 has been found to be involved in ER stress-induced cell death^21^. Theoretically, this common activation of the UPR between the different compounds suggests that the necessary mediators for cell death induced by these agents could be shared between them. However, our screens indicated that each of these agents requires different and distinct genes to cause cell death in HAP1 cells. Our findings showed that the identified genes mediate cytotoxicity in an agent-specific manner, and the mediation occurs upstream of activation of the UPR. This is supported by the facts that thapsigargin, tunicamycin, and brefeldin A all employ different and distinct mechanisms to induce ER stress and that the identified genes have known functions in processes that occur upstream of the UPR. Tunicamycin inhibits N-linked glycosylation of proteins in the ER to induce stress^5^. As shown by our current screen and the previous screen, MFSD2A is required for transporting tunicamycin into the cell, where it can induce cytotoxicity upstream of the UPR^18^. With regard to brefeldin A, ER stress is induced by the collapse of the Golgi into the ER^9^. As shown in our screen and the previous screen, brefeldin A requires ARF4, a small guanidine nucleotide-binding protein that is important for Golgi–ER retrograde vesicular trafficking, to cause brefeldin A-induced cell death^6^. ARF4 acts upstream of Golgi collapse and subsequent UPR activation to assist in retrograde trafficking from the Golgi to the ER. Finally, thapsigargin inhibits the SERCA pump and upsets calcium homeostasis in the ER, which results in ER stress and activation of the UPR if not resolved^10,11^. As shown in our screen, thapsigargin requires SEC24A, a component of COPII ER–Golgi trafficking machinery^12–14^, to cause cell death. Our data show that SEC24A acts upstream of the UPR to cause thapsigargin-induced cell death. The basis of this interpretation is that, when treated with thapsigargin, SEC24A mutant cells only slightly activate the UPR compared to HAP1 WT cells. Together, these data indicate that the genes identified in our screens act upstream of the UPR in an agent-specific manner to induce cell death.

A few scenarios can be envisioned for the role of SEC24A in thapsigargin-induced cytotoxicity, and all of them will require further studies to elucidate. First, given that the SEC24A protein has a known role in COPII-dependent ER–Golgi trafficking of proteins^12–14^, the results of our thapsigargin screen could suggest that SEC24A transports specific proteins that are necessary for thapsigargin-induced cytotoxicity. The fact that no other genes were found in the screen suggests redundancy in the SEC24A-dependent cargo that results in thapsigargin-induced cell death. Of the known SEC24A cargoes, a potential candidate for our screen is ATF6, one of the three effector proteins of the UPR^10^.

ATF6 must be secreted from the ER to the Golgi in order to become activated and to assist in the UPR^10^. ATF6 has been shown to induce apoptosis as a result of ER stress in mouse myoblasts^22^. Additionally, through a partner protein, ATF6 has been shown to be dependent on SEC24A for ER to Golgi translocation^23^. Thus ATF6 may be one of the SEC24A-dependent cargo involved in cell death induction after thapsigargin treatment.

A second scenario is based on the ability of SEC24A to induce cell death in an agent-specific manner. This suggests that thapsigargin’s mechanism of action may influence how SEC24A mediates thapsigargin-induced cytotoxicity. Thapsigargin inhibits the SERCA pump, which results in ER calcium depletion and dysregulation of cellular calcium homeostasis^10,11^. With regard to our results, SEC24A may influence calcium homeostasis either directly or indirectly. In this scenario, SEC24A mutants may confer resistance to thapsigargin-induced cell death because they are able to prevent the onset of the calcium imbalance, or they are able to elicit a response that can correct thapsigargin-induced calcium imbalances in the cell.

In a final scenario, SEC24A might serve as a signaling protein for cell death induced by thapsigargin. This would be analogous to the role of death receptor 5 in caspase-8-mediated apoptosis due to unresolved ER stress^24^. To our knowledge, SEC24A has not been identified as a signaling protein in previous work.

In summary, our positive selection screens against the ER stress inducers thapsigargin, tunicamycin, and brefeldin A identified different genes as being necessary for cell death induced by each agent. The thapsigargin screen identified a novel necessary mediator of thapsigargin-induced cytotoxicity, SEC24A. Further experiments showed that the behavior of SEC24A as a mediator of cell death is specific to thapsigargin and acts upstream of the UPR to facilitate thapsigargin-induced cell death. Our identification of a previously unknown role for SEC24A as an essential mediator of thapsigargin-induced cytotoxicity opens a new avenue for investigating how diverse forms of ER stress specifically activate the UPR and trigger cell death.

## Materials and methods

### Cell lines and vectors

Materials were obtained from the following sources: HAP1 wild-type (WT) cells were from Horizon Discovery (Cambridge, UK); HEK293FT cells were kindly provided by Michael Cole (Geisel School of Medicine at Dartmouth, Lebanon, NH); and Human GeCKO v2 Library (1 plasmid system), lentiCRISPRv2, pMD2.G, and psPAX2 were obtained from Addgene (Cambridge, MA, USA*)*.

### Cell culture and maintenance

HAP1 WT cells were cultured in Iscove’s Modified Dulbecco’s Medium (IMDM; Sigma-Aldrich, St. Louis, MO, USA) supplemented with 10% fetal bovine serum (FBS; Hyclone, Logan, UT, USA), 4 mM L-glutamine, and 1× penicillin/streptomycin (Corning, Corning, NY, USA). HEK293FT cells were cultured in IMDM supplemented with 10% FBS, 6 mM L-glutamine, 1× penicillin/streptomycin, and 1× non-essential amino acids (Sigma-Aldrich) for the first 24 h following thawing. After 24 h, G418 (Sigma-Aldrich) was added to the growth medium to a final concentration of 500 µg/mL. Both cell lines were maintained at 37 °C, 5% CO_2_.

### Amplification of pooled lentiviral library

GeCKO v2 libraries A and B (100 ng each) were electroporated separately into Endura Electrocompetent cells (Lucigen, Middleton, WI, USA) per the manufacturer’s protocol. Each library was electroporated 4 times, and recovery reactions were rotated at 250 rpm for 1 h at 37 °C. Transformations were pooled, and a dilution plate was prepared for each library to calculate transformation efficiency and to ensure that each sgRNA construct was represented at least 50× in the amplified libraries. Aliquots (400 µL) of the pooled reactions were plated on 20 × 10 cm^2^ LB-Ampicillin (100 µg/mL) (Sigma-Aldrich) agar plates. Plates were incubated at 32 °C for 14 h. Colonies were harvested by washing each plate with 500 µL of LB medium twice using a cell scraper and pooled. Endotoxin Free Plasmid Maxi Kit (Qiagen, Germantown, MD, USA) was used to isolate DNA per the manufacturer’s protocol.

### Lentivirus production in HEK293FT cells

HEK293FT cells were seeded 24 h before transfection in 10-cm plates to allow for ~60% confluence on the day of transfection. Medium containing G418 was removed 30 min before transfection and replaced with 10 mL of IMDM supplemented with 10% FBS, 6 mM L-glutamine, 1× penicillin/streptomycin, 1× non-essential amino acids, and 2 mM caffeine (Sigma-Aldrich). Transfection reactions were prepared in 500 µL of IMDM supplemented with 6 mM L-glutamine, 1× penicillin/streptomycin, and 1× non-essential amino acids (transfection medium), with 5 µg library DNA, 3 µg psPAX2, and 2 µg pMD2.G, and gently vortexed. LipoD293 (Signagen, Rockville, MD, USA) (30 μL) was added to 500 µL of transfection medium and gently vortexed. This diluted LipoD293 was immediately added to the DNA mixture, gently vortexed, and incubated for 10 min at room temperature. The LipoD293/DNA mixture was added slowly to the cells and incubated for 24 h at 37 °C, 5% CO_2_. The medium was then replaced with IMDM supplemented with 2% FBS, 6 mM L-glutamine, 1× penicillin/streptomycin, 1× non-essential amino acids, and 2 mM caffeine and incubated for an additional 24 h. Lentiviral particles were then harvested by filtration through a 0.45-µm filter.

### Lentivirus titering and transduction in HAP1 WT cells

HAP1 WT cells were seeded 24 h before transduction in 12-well plates (Corning) to allow for ~60% confluence on the day of transduction. Serial dilutions (10-fold) of lentiviral particles, ranging from 10^−1^ to 10^−3^ were prepared in growth medium, and 500 µL of the diluted lentiviral particles were added to the cells. Medium (500 µL) containing 16 μg/mL protamine sulfate (MP Biomedicals, Santa Ana, CA, USA) was added to each of the wells. After 24 h, virus and protamine sulfate containing medium was replaced with growth medium. After 24 h, selection was initiated by addition of puromycin (Sigma-Aldrich) (1.5 μg/mL final concentration) to the growth medium. Medium was replaced every 48 h with fresh medium containing 1.5 µg/mL puromycin thrice. The cells were under puromycin selection for a total of 5 days. The lentivirus titer was determined using the formula:

Titer = ((number of cells remaining after puromycin selection) × (dilution factor))/(volume of diluted lentivirus used) = transduction units/mL = TU/mL.

### Transduction of HAP1 WT cells to generate GeCKO A and GeCKO B libraries in HAP1 cells

HAP1 WT cells were seeded in 15 cm plates 24 h before transduction to allow for ~60% confluence on the day of transduction. Cells were transduced with lentivirus at an MOI of 0.3 in the presence of 8 µg/mL protamine sulfate. The total number of cells that were transduced ensured 400× representation of each single-guide RNA (sgRNA) construct in each of the GeCKO v2 libraries. The medium was replaced with growth medium after 24 h of incubation at 37 °C, 5% CO_2_, and 24 h later, 1.5 µg/mL puromycin was added for selection of transduced cells. Cells were grown under selection for 5 days with medium renewal every 48 h. CRISPR/Cas9-modified HAP1 cells were expanded, and to ensure maintenance of adequate representation, each library was frozen down at 1200–1600× representation of each sgRNA construct in the libraries. Libraries were stored in liquid nitrogen until use. Genomic DNA was extracted from 4 × 10^7^ cells using the Blood and Cell Culture DNA Midi Kit (Qiagen) to check the libraries for sgRNA representation.

### Cytotoxicity assays for ER stress inducers in HAP1 WT cells

Tunicamycin, thapsigargin, and brefeldin A (Sigma-Aldrich) were used as ER stress inducers. HAP1 WT cells were seeded in 6-well plates (Corning) 24 h before stress induction to allow for ~60% confluence on the day of treatment. HAP1 WT cells were treated with a range of concentrations of ER stressors, and after 4 days, cell viability was determined using trypan blue and a hemocytometer to count live cells. Cytotoxicity curves were generated for each ER stress inducer, and the concentration of each agent at which ~1% of the cells survived the stress was determined. These concentrations—thapsigargin, 0.062 µg/mL; tunicamycin, 0.2 µg/mL; and brefeldin A, 0.045 µg/mL—were selected for screens in HAP1 CRISPR/Cas9 libraries and were used in all experiments reported herein.

### Positive selection screens using ER stress inducers in HAP1 CRISPR libraries

HAP1 CRISPR library cells (1.5 × 10^7^) were seeded on 2 × 15 cm^2^ plates for each library (A and B libraries). Cells were treated 24 h later with medium containing ER stress inducer. Thereafter, cells were trypsinized and treated with medium containing fresh ER stress inducer every 3 days for a total of 12 days. Surviving cells were expanded in medium without ER stressor and genomic DNA was extracted from 4 × 10^7^ cells using the Blood and Cell Culture DNA Midi Kit (Qiagen).

### Genomic DNA sequencing

To amplify sgRNA regions of interest from the genomic DNA, 3 rounds of PCR were performed. For the first PCR, 22 amplification cycles were run for 18 separate 100 µL reactions, each with 5 µg of genomic DNA, using Herculase II Fusion DNA Polymerase (Agilent, Santa Clara, CA, USA) and PCR1 primers shown in Table 1. Products from the first PCR were purified using a QIAquick PCR Purification Kit (Qiagen). In the second PCR, which was used to add Illumina adaptors and barcodes, 22 amplification cycles were run for 12 separate 100 µL reactions, each using 5 µL of pooled sample from the first PCR, Herculase II Fusion DNA Polymerase, and primers containing Illumina adaptors and barcodes^25^. Each reaction contained a unique forward primer, while reverse primers were used to barcode different sets of samples. Products from the second PCR were purified using a QIAquick PCR Purification Kit (Qiagen). For the third PCR, 22 cycles were run for 100 µL reactions each with 150 ng of purified PCR product from the second PCR using PCR3 primers shown in Table 1. The PCR products were electrophoresed on 2.5% LMP Agarose (Promega, Fitchburg, WI, USA) gels. The desired amplicons were isolated from the gels using the Gel Extraction Kits (Qiagen), and these products were further purified using the QIAquick PCR Purification Kits. Samples were sequenced on a HiSeq 2500 Illumina platform.

**Table 1:** Sequences of primers and oligonucleotides

Identification	Sequence (5’→3’)
Amplification primers for deep sequencing	
PCR1 forward primer	AATGGACTATCATTGAAAGTATTTCG
PCR1 reverse primer	TCTACTATTCTTTGATGTGCGCTCTG
PCR3 forward primer	AATGATACGGCGACCACCGAGATC
PCR3 reverse primer	CAAGCAGAAGACGGCATACGAGAT
U6 forward primer	CGTGACGTAGAAAGTAATAATTTCTTGGG
sgRNA sequences	
MFSD2A sgRNA 1	GAACATGGTGAGAGCCGAGT
MFSD2A sgRNA 1	GGAAACAAGGCGTGTCTGCT
ARF sgRNA 1	TCTGCTTCTTGCCAAATAGT
ARF sgRNA 2	CTAGTTGGATTGGATGCTGC
SEC24A sgRNA 1	AGTAGTTACGACGAGATTGA
SEC24A sgRNA 2	GCAGGACCACTATGAAGCGA
SEC24A sgRNA 3	GGGATGATGCACGAGGACAA
PNPLA8 sgRNA 1	ATCCGAATTCTCTCAATTGA
EGFP sgRNA	AGCTGGACGGCGACGTAAA
Amplification primers for Sanger sequencing	
SEC24A forward primer	CAAGGATACAATTTCCAGCTTCCAG
SEC24A reverse primer	TATATTGCCAATTCAAAGGTGGTGG
PNPLA8 forward primer	TCAGGCTGTCAGCAAAGACA
PNPLA8 reverse primer	AGCTTTGGGTGTTATCCCTCT

### Data processing and analysis

Model-based analysis of genome-wide CRISPR/Cas9 knockout (MAGeCK) was used to process and analyze data from the HiSeq sequencing platform^16^. MAGeCK was run using default parameters with the Human GeCKO v2 combined library (Addgene) as a reference. Criteria for determining true hits from the MAGeCK results were multiple sgRNAs per gene and significant and consistent enrichment of two or more sgRNAs targeting the particular gene. Data files generated using MAGeCK are presented as Supplemental Data Sets and represent the following: thapsigargin and tunicamycin screen normalized counts (Supplementary Data Set 1), thapsigargin screen gene summary (Supplementary Data Set 2), tunicamycin screen gene summary (Supplementary Data Set 3), brefeldin A normalized counts (Supplementary Data 4), and brefeldin A gene summary (Supplementary Data Set 5). Tables 2, 3, and 4 show positive selection screen data and are derived from Supplementary Data Sets 2, 3, and 5, respectively.

**Table 2:** Ranking of genes in positive selection thapsigargin screen using MAGeCK

Rank	Gene	Number of sgRNAs^a^	RRA score^b^	*p* ^c^	FDR	Good sgRNAs	LFC^d^
1	SEC24A	6	8.14E−15	2.27E−07	0.00495	5	2.3673
2	PNPLA8	6	1.74E−06	3.86E−06	0.042079	2	−5.8655
3	C12orf40	6	2.52E−05	5.02E−05	0.364686	1	−7.0267
4	PSEN1	6	7.56E−05	9.60E−05	0.523515	1	−6.335
5	ALG6	6	0.00012606	0.00014236	0.620792	1	−7.2067
6	STK17B	6	0.00017648	0.00020321	0.738449	1	−6.8258
7	DPH5	6	0.00022689	0.00024181	0.753182	1	−7.1674
8	ZNF804B	6	0.00027731	0.00029267	0.797649	1	−6.7912
9	MARCH10	6	0.00032772	0.00034308	0.810396	1	−7.3341
10	LNP1	6	0.00035713	0.00037169	0.810396	1	−6.5594

*FDR* false discovery rate

^a^Number of sgRNAs refers to number of sgRNAs targeting the gene

^b^RRA score is derived from a robust ranking aggregation (RRA) algorithm that determines the degree of consistency seen in the enrichment of multiple sgRNAs targeting a particular gene, compared to sgRNAs targeting other genes

^c^*p* Value is generated for these RRA scores

^d^LFC is log fold change of sgRNAs

**Table 3:** Ranking of genes in positive selection tunicamycin screen using MAGeCK

Rank	Gene	Number of sgRNAs^a^	RRA score^b^	*p* ^c^	FDR	Good sgRNAs	LFC^d^
1	MFSD2A	6	6.27E−26	2.27E−07	0.00495	6	11.262
2	TBX5	6	8.40E−05	0.00016734	0.905049	2	−5.0824
3	AGPHD1	3	0.00021431	0.00023182	0.905049	1	−6.6886
4	C1orf95	5	0.00027313	0.00032083	0.905049	1	−6.3316
5	ALDH4A1	6	0.00027733	0.00036397	0.905049	1	−6.3373
6	TMED7-TICA	5	0.00027733	0.00045343	0.905049	1	−7.1405
7	FBXO42	6	0.0005294	0.00061282	0.905049	1	−7.097
8	UROS	6	0.0005798	0.00066323	0.905049	1	−7.2471
9	CHURC1	6	0.0006302	0.00070501	0.905049	1	−7.4473
10	APOBEC1	6	0.00068061	0.00075224	0.905049	1	−6.9459

*FDR* false discovery rate

^a^Number of sgRNAs refers to number of sgRNAs targeting the gene

^b^RRA score is derived from a robust ranking aggregation (RRA) algorithm that determines the degree of consistency seen in the enrichment of multiple sgRNAs targeting a particular gene, compared to sgRNAs targeting other genes

^c^*p* Value is generated for these RRA scores

^d^LFC is log fold change of sgRNAs

**Table 4:** Ranking of genes in positive selection brefeldin A screen using MAGeCK

Rank	Gene	Number of sgRNAs^a^	RRA score^b^	*p* ^c^	FDR	Good sgRNAs	LFC^d^
1	ARF4	6	1.05E−19	2.29E−07	0.00495	6	10.185
2	OGFRL1	5	2.58E−05	3.50E−05	0.378713	2	−4.953
3	hsa-mir-4676	3	0.00013943	0.00015396	0.725548	1	−7.014
4	hsa-mir-106a	4	0.00014459	0.00016952	0.725548	1	−5.515
5	CCDC33	6	0.00015492	0.00018233	0.725548	1	−4.149
6	EDN3	5	0.00028401	0.00029488	0.725548	1	−3.398
7	ANKRD35	6	0.00040275	0.00042939	0.725548	1	−4.256
8	NonTargeting	1	0.00042864	0.00043443	0.725548	1	6.0675
9	NonTargeting	1	0.00048029	0.00048521	0.725548	1	5.6036
10	ACAP1	6	0.00052665	0.0005424	0.725548	1	−5.284

^a^Number of sgRNAs refers to number of sgRNAs targeting the gene

^b^RRA score is derived from a robust ranking aggregation (RRA) algorithm that determines the degree of consistency seen in the enrichment of multiple sgRNAs targeting a particular gene, compared to sgRNAs targeting other genes

^c^*p* Value is generated for these RRA scores

^d^LFC is log fold change of sgRNAs

### Phenotypic validation of candidate genes from screens

sgRNA sequences for genes identified as hits were obtained from Addgene. sgRNAs that showed the highest count numbers in our screens after MAGeCK analysis were used for validation (Table 1). sgRNAs were cloned into the lentiCRISPRv2 (Addgene) backbone as previously described^25^ to generate specific lentiCRISPR plasmids. Using the U6 forward primer, the plasmids were Sanger sequenced to ensure that the sgRNAs were inserted in the lentiCRISPR plasmids correctly. Turbofectamine 8.0 (Origene, Rockville, MD, USA) was used to transfect HAP1 cells with the lentiCRISPR plasmids per the manufacturer’s protocol to generate stable polyclonal mutant cell lines. Complete IMDM containing 1.5 µg/mL puromycin was used for a total of 5 days to select for transfected cells with refreshment of puromycin every 48 h. Monoclonal cell lines were isolated using serial dilution in 96-well plates and kept under thapsigargin stress (0.062 µg/mL) (Corning). For phenotypic validations, cell lines were seeded in 12-well plates, and 24 h later, at ~60% confluence, the cells were treated with thapsigargin (0.062 µg/mL), tunicamycin (0.2 µg/mL), or brefeldin A (0.045 µg/mL). Cell viability was determined after 3 days using trypan blue and counting live cells on a hemocytometer.

### Immunoblotting and blot analysis

Whole-cell lysates from HAP1 WT and mutant monoclonal cell lines were isolated using PhosphoSafe Extraction Reagent (EMD Millipore Novagen, Burlington, MA, USA) per the manufacturer’s protocol. Lysates were run on 12% sodium dodecyl sulfate polyacrylamide gels and visualized by western blotting using the following antibodies: SEC24A antibody (#9678), CCAAT/enhancer-binding protein homologous protein (CHOP) (L63F7) (#2895), activating transcription factor 4 (ATF-4) (D4B8) (#11815), anti-rabbit IgG, horseradish peroxidase (HRP)-linked antibody (#7074), anti-mouse IgG, HRP linked antibody (#7076) (Cell Signaling Technology, Danvers, MA, USA), and PNPLA8 antibody (#PA5-32006) (Invitrogen, Waltham, MA, USA).

The blot was analyzed and quantified using ImageJ (NIH). A region of interest (ROI) was generated using the largest band, and this ROI was used to measure all band intensities. Each band was measured five times. Background intensity values were obtained using the ROI in five randomly selected areas of the blot that did not have any distinct bands. Average background intensity was calculated and subtracted from each band value, and data were plotted using the GraphPad Prism software.

### Genotyping of monoclonal cell lines

Genomic DNA was isolated from HAP1 WT and mutant monoclonal cell lines using the Blood and Cell Culture DNA Midi Kit (Qiagen). Primers (Table 1) were designed to amplify ROIs in the SEC24A and PNPLA8 genes where CRISPR/Cas9 modifications were likely to have occurred based on sgRNA sequences used to generate the cell lines. KAPA HiFi HotStart DNA Polymerase (KAPA Biosystems, Cape Town, South Africa) was used to generate amplicons that were analyzed for correct size on low-melting point agarose gels. Amplicons were gel extracted using the Gel Extraction Kit (Qiagen) and Sanger sequenced using the appropriate SEC24A and PNPLA8 sequencing primers (Table 1).

## Supplementary information

Supplementary Materials

Supplemental Data Set 1

Supplemental Data Set 2

Supplemental Data Set 3

Supplemental Data Set 4

Supplemental Data Set 5
