# Supplementary material for: SEC24A identified as an essential mediator of thapsigargin-induced cell death in a genome-wide CRISPR/Cas9 screen
Source: Cell Death Discov. 2018 Dec 18;4:115. doi: 10.1038/s41420-018-0135-5 (PMC6299087; doi:10.1038/s41420-018-0135-5)
Supplement: Supplementary file 1 — Supplementary Materials [file 41420_2018_135_MOESM1_ESM.docx]

**Supplementary Materials:**

1. **B. C.**

**Supplementary Fig. 1: Cytotoxicity curves for the ER stress inducing agents thapsigargin, tunicamycin, and brefeldin A in HAP1 WT cells.** HAP1 WT cells were treated with the ER stress inducers, thapsigargin **(A)**, tunicamycin **(B)**, and brefeldin A **(C)**, for 4 days to determine cytotoxic concentrations. Cell survival was determined using vital staining with trypan blue, and is shown as a percentage relative to untreated control cells.

1. **C.**

**HAP1 WT:**

**PNPLA8 mutant 1:**

**PNPLA8 mutant 2:**

**AAAGGGAGAGGAATCCCAATTTTCATAATTGAAGATGGAGGA**

**AAAGGGAGAGGAATCCCAATTTTCATAATTGAAGATGGAGGA**

**AAAGGGAGAGGAATCCCAATTTTCATAATTGAAGATGGAGGA**

**HAP1**

**WT**

**PNPLA8**

**mutant**

**M_r_**

**(kDa)**

**2**

**1**


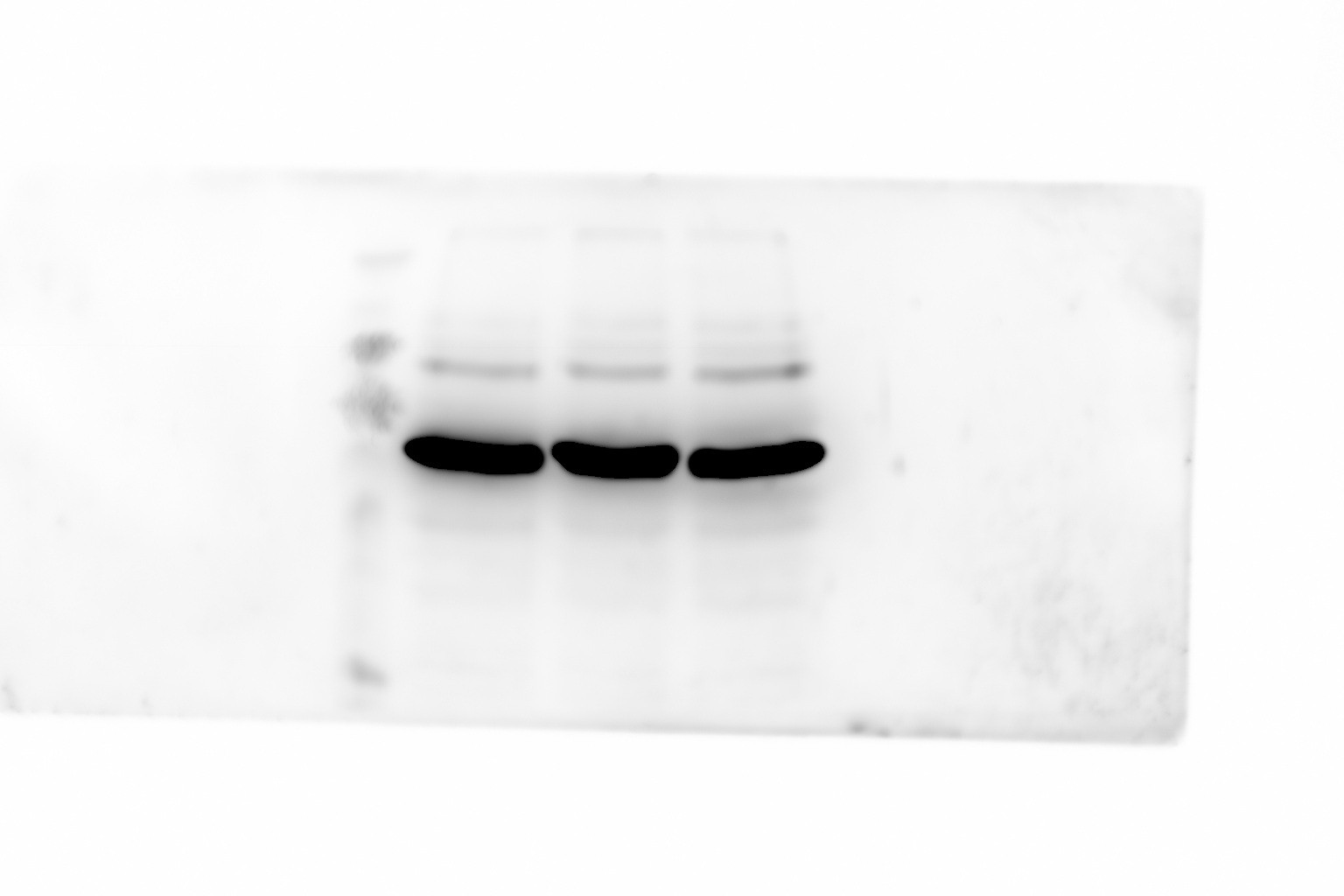


**88**

**PNPLA8**

**Supplementary Fig. 2: Validation of PNPLA8 candidate gene from thapsigargin screen.** Two monoclonal PNPLA8 mutant cell lines generated from 1 sgRNA (PNPLA8 sgRNA 1 in **Table 1**) were treated with thapsigargin. **(A)** After 3 days of treatment with 0.062 µg/mL thapsigargin, resistance to thapsigargin-induced cell death compared to HAP1 WT cells was determined. Cell survival was assessed in PNPLA8 mutant cell lines and HAP1 WT cells using trypan blue. N = 6. **(B)** Western blot showing PNPLA8 protein in HAP1 WT cells and in PNPLA8 mutants 1 and 2. **(C)** Genotyping PNPLA8 mutant monoclonal cell lines. The underlined regions represent the location in the PNPLA8 gene that was targeted for mutation by PNPLA8 sgRNA 1.
